# Emergence and Comparative Analysis of *Candidozyma auris* Versus *Candida* spp. Candidemia in a Romanian Tertiary Hospital: A 7-Year Study on Resistance, Mortality and Independent Prognostic Factors

**DOI:** 10.3390/jof12070482

**Published:** 2026-07-01

**Authors:** Sebastian George Smadu, Simona Camelia Tetradov, Corneliu Petru Popescu, Maria Nica, Corina Oprisan, Luminita Ene, Simin Aysel Florescu

**Affiliations:** 1 Faculty of Medicine, “Carol Davila” University of Medicine and Pharmacy, 37 Dionisie Lupu Street, 020021 Bucharest, Romania; sebastian-george.smadu@drd.umfcd.ro (S.G.S.); corneliu.popescu@umfcd.ro (C.P.P.); maria.nica@umfcd.ro (M.N.); corina.barbu@drd.umfcd.ro (C.O.); simin.florescu@umfcd.ro (S.A.F.); 2 “Victor Babes” Clinical Hospital for Infectious and Tropical Diseases, 281 Mihai Bravu Street, 030303 Bucharest, Romania; luminita.ene@spitalulbabes.ro

**Keywords:** invasive candidiasis, *Candidozyma* auris, candidemia, *Candida*, antifungal resistance, healthcare associated infection, risk factors, mortality, prognostic factors

## Abstract

Background: Candidemia remains a major cause of morbidity and mortality among hospitalized patients. The emergence of *Candidozyma auris* has added further complexity due to its persistence in healthcare settings and its high rates of antifungal resistance. Comparative real-world data between *Candidozyma auris* and *Candida* spp. candidemia remain limited. Methods: We conducted a retrospective cohort study that included adult patients with candidemia, admitted to a tertiary infectious diseases hospital in Romania between August 2018 and August 2025. Risk factors, including medical history, previous hospitalizations, clinical characteristics, laboratory parameters, antifungal susceptibility patterns, treatment, and outcomes, were compared in patients with *Candidozyma auris-* and *Candida* spp.-positive blood cultures. Overall survival and prognostic factors were evaluated using univariable and multivariable Cox-proportional hazards models. Results: Sixty-one patients with candidemia were included; out of them, 24 (39.3%) had *Candidozyma auris*-positive blood cultures. *Candidozyma auris* infections, which emerged later during the study period, occurred after a significantly longer period of hospitalization compared with *Candida* spp. candidemia (median 52.5 vs. 20 days, *p* < 0.001). Azole resistance was almost universal among *Candidozyma auris* isolates (95.8%), whereas *Candida* species displayed significantly lower resistance rates and a broader susceptibility spectrum (*p* < 0.001). Inflammatory markers were comparable between groups; however, *Candidozyma auris* candidemia was associated with lower neutrophil counts and lower neutrophil-to-lymphocyte ratios at diagnosis (*p* = 0.020). Persistent candidemia at day 7 occurred more frequently in *Candidozyma auris* infections (6 vs. 2 patients; *p* = 0.05) and was universally fatal. Overall, in-hospital mortality was high (70.5%) and did not differ between *Candidozyma auris* and *Candida* spp. candidemia. In multivariable analysis, thrombocytopenia < 100,000/μL was independently associated with mortality (HR 2.34, 95% C.I. 1.20–4.56; *p* = 0.012). Conclusions: In this 7-year study at a Romanian tertiary center, *Candidozyma auris* emerged as a major healthcare-associated pathogen affecting patients with significantly prolonged hospitalization (median 52.5 days). Despite near-universal azole resistance (95.8%), mortality was exceptionally high (70.5%) and was comparable between groups, with pathogen type not independently associated with outcome after multivariable adjustment. Moderate thrombocytopenia (<100,000/μL) and persistent candidemia identified patients at particularly high risk of death, underscoring the need for early risk stratification, optimized antifungal management, and enhanced diagnostic vigilance, both in intensive care and general wards.

## 1. Introduction

The epidemiology of candidemia has changed substantially in the recent years, driven by the emergence of *Candidozyma auris* and further influenced by the COVID-19 pandemic. An important shift from *Candida albicans* to non-albicans species has been observed, along with increasing rates of fluconazole resistance and even echinocandins [1,2].

*Candidozyma auris*, first reported in Japan in 2009, initially emerged as an uncommon pathogen causing sporadic fungal infections. Within a decade, it became a globally distributed pathogen, characterized by its primary ability to cause outbreaks in healthcare facilities, due to its resilient persistence on environmental surfaces. These features, along with the increasing incidence worldwide, led the WHO to include *Candidozyma auris* on the fungal pathogen priority list [3,4].

Risk factors associated with candidemia can be divided into two major categories. The first one includes healthcare-associated exposures, such as prolonged hospitalization, admission into intensive care units, presence of catheters, broad spectrum antibiotic use, total parenteral nutrition or colonization at multiple anatomical sites. The second comprises conditions that impair host immunity, including hematological malignancies, neoplasia, solid organ or stem cell transplantation, and the use of immunosuppressive therapy [5].

*Candida auris*, recently reclassified in 2023 and renamed *Candidozyma auris*, is a resilient yeast capable of colonizing a wide range of hospital surfaces. The major pathogenic feature is biofilm formation mediated by enhanced adhesion to abiotic surfaces and the production of an extracellular matrix that limits antifungal penetration, along with antifungal resistance and reduced susceptibility to common disinfectants. These contribute to its marked persistence and the high levels of transmission within healthcare settings. Patient colonization has been documented to occur within the first hours following exposure to contaminated surfaces, whereas progression to infection from colonization state in susceptible patients appears within subsequent days, after approximately 48 h [6,7,8].

*Candida* spp. causing bloodstream infections most commonly originate from endogenous colonization of the gastrointestinal tract, with invasion facilitated by the disruption of the mucosal barrier and invasive medical devices. In contrast, *Candidozyma auris* has consistently been associated with sustained environmental persistence and healthcare-associated transmission, which may facilitate its spread within healthcare settings [9].

The aim of the present study was to characterize and compare clinical characteristics, laboratory findings, antifungal susceptibility profiles, and clinical outcomes among patients diagnosed with invasive candidiasis, focusing on the differences between *Candidozyma auris* and *Candida* spp. infections.

As our aim was to differentiate the emerging, environmentally acquired pathogen *Candidozyma auris* from conventionally acquired candidemia, *Candida* species were evaluated as a unified comparator group. While this pragmatic categorization aligns with their shared, predominantly endogenous (gastrointestinal) origin—distinguishing them from the healthcare-associated, environmental transmission of *C. auris*—it acknowledges but does not capture the substantial inter-species variability regarding virulence and antifungal resistance profiles [10,11,12].

## 2. Materials and Methods

### 2.1. Study Design and Population

We conducted a retrospective observational study on a cohort that included all patients with candidemia admitted to “Dr. Victor Babes” Clinical Hospital for Infectious and Tropical Diseases (VBH), Bucharest, Romania, during the 7-year period (between 1 August 2018 and 1 August 2025). VBH is a tertiary-care infectious diseases hospital, comprising 490 beds and including a dedicated intensive care unit, and serves as one of the two referral centers for infectious diseases in Bucharest.

Inclusion criteria were hospitalized adult patients (>18-year-old) with Candidemia, defined as at least one positive blood culture for *Candida* spp. Culture obtained from the central line was excluded to reduce risk of colonization and in order to standardize case definition.

This study was conducted in accordance with the Declaration of Helsinki and approved by the Institutional Ethics Committee of “Dr. Victor Babes” Clinical Hospital for Infectious and Tropical Diseases, Bucharest, under approval number 4809/17 March 2026. All patients provided written informed consent for the use of anonymized clinical data at hospital admission as part of routine administrative procedures.

### 2.2. Data Collection and Statistical Analysis

Medical records were systematically reviewed for demographic data, underlying condition, risk factors, primary infection source, laboratory parameters and outcomes. Comorbidities assessed included diabetes mellitus, malignancies, hematologic conditions, transplant history, and other significant medical conditions. Bedridden status was defined as continuous immobility or confinement to bed for at least 7 consecutive days prior to the diagnosis of candidemia. Corticotherapy was defined as systemic corticosteroid treatment administered for at least 7 days consecutive prior to diagnosis, at a minimum dose of 0.5 mg/kg/day of prednisolone or equivalent. Healthcare-associated factors included previous hospital admissions, presence of central venous catheters, urinary catheters, nasogastric tubes, mechanical ventilation, recent surgery (past 30 days), and antibiotic use in the past 30 days.

Based on the *Candida* species identified, patients were stratified into two groups: *Candidozyma auris* and *Candida* spp.

All the data from the study were analyzed using IBM SPSS Statistics 25 (IBM Corp., Armonk, NY, USA). Qualitative variables were written as counts or percentages and were compared between groups. Z-tests with Bonferroni correction were used to further evaluate the results obtained in the contingency tables. Quantitative variables were presented as means with standard deviations or medians with interquartile ranges. Normality of the quantitative variables was assessed using the Shapiro–Wilk Test. Quantitative independent variables with non-parametric distribution were tested between groups using the Mann–Whitney U Test, and those with normal distribution were tested between groups using the Student T-Test/Welch T-Test (according to the equality of variances between groups observed using the Levene test).

In each of the analyzed groups (*Candida* spp. and *Candidozyma auris* group), evolution of laboratory parameters was measured using the Paired-Samples T-Test (for variables with normal distribution) or the Wilcoxon Test (for variables with non-parametric distribution). Continuous variables were compared using the Mann–Whitney U test; categorical variables were compared using Fisher’s exact test.

Overall survival was defined as the time from the first positive blood culture to hospital discharge or death. Survival distributions were estimated using Kaplan–Meier curves and reported as median survival with 95% confidence intervals and compared between groups using the log-rank test. Data were censored at the time of last clinical contact, including follow-up visits.

Univariable and multivariable Cox-proportional hazard models were used to estimate the effect of investigated factors on mortality. Models were tested for significance and goodness-of-fit; proportional-hazards assumptions were tested using R software (The R project for Statistical Computing) version 4.4.0, using survival and survminer packages. The performance of the prediction was calculated as hazard ratios with 95% confidence intervals.

The threshold considered for the significance level for all tests was α = 0.05.

### 2.3. Laboratory Parameters

Laboratory parameters were documented at the time when invasive fungal infection was clinically suspected and confirmed by blood culture, as well as on day 7 after diagnosis. This included complete blood count (white blood cells, hemoglobin, platelets), calculated using an automated hematology analyzer (DxH 900, Beckman Coulter, Brea, CA, USA) and renal function (Creatinine), measured by an enzymatic method on a Cobas c303 analyzer (Roche Diagnostics, Mannheim, Germany). Inflammatory markers were C-reactive protein (CRP) with an upper normal limit of 0.5 mg/dL measured by immunonephelometry (Cardio Phase^®^ hsCRP, Siemens, Munich, Germany, Catalog no. OQIY21) and Procalcitonin (PCT) with an upper normal limit of 0.05 ng/dL measured by chemiluminescent microparticle immunoassay (Alinity, Abbott; PCT Reagent Kit, Chicago, IL, USA, catalog no 01R1822). These tests included the hospital’s protocols of a standard evaluation of patients with suspected infection. The neutrophil-to-lymphocyte ratio (NLR) was calculated by dividing the absolute neutrophil count by the absolute lymphocyte count.

### 2.4. Microbiological Methods

All fungal identification was performed using automated systems based on biochemical profiling (VITEK 2C-bioMérieux, Marcy-l’Étoile, France), as well as mass spectrometry (MALDI-TOF-BRUKER, Billerica, MA, USA). Antifungal susceptibility testing was conducted according to Clinical and Laboratory Standards Institute (CLSI) criteria between 2018 and 2019 and the European Committee on Antimicrobial Susceptibility Testing (EUCAST) standards during the 2020–2025 period. Minimum inhibitory concentrations (MICs) were determined using VITEK 2C AST-Y08 cards (bioMérieux) for *Candida* spp. and the broth microdilution method (Micronaut AM, Bruker, Billerica, MA, USA) for *Candidozyma auris* isolates.

For the purpose of this analysis, and to provide clinically actionable susceptibility data, all MIC values were retrospectively interpreted according to EUCAST clinical breakpoints version 12.0 (effective 26 June 2025) which established species-specific breakpoints for *Candidozyma auris* [13]. Susceptibility was categorized as susceptible (S), intermediate (I), or resistant (R) according to EUCAST definitions [13]. Resistance was further classified into three major antifungal drug classes to facilitate clinical interpretation: azole resistance (resistant to fluconazole and/or voriconazole), polyene resistance (resistant to amphotericin B), and echinocandin resistance (resistant to anidulafungin, caspofungin, and/or micafungin). When MIC data for the primary agent within a drug class were unavailable, interpretation was based on an alternative agent from the same class.

### 2.5. Outcome

The study aimed to record, when available, a day-7 assessment, including laboratory parameters, repeat blood cultures to confirm fungal clearance, and risk-stratified investigations.

The following factors were assessed in follow-up evaluation: the hospitalization site [general ward versus intensive care unit (ICU)], duration of hospitalization, persistent candidemia (defined as positive blood cultures after 7 days), and concomitant bacterial infection.

Primary outcome was overall survival, defined as time from positive blood culture to discharge or death, and all-cause in-hospital mortality.

## 3. Results

### 3.1. Baseline Characteristics

The study group included 61 patients with candidemia, with a median age of 66 years (IQR: 54–73.5, 95% C.I. = 59.25–66.85), and most of them were male (45/61, 73.8%). The diagnosis at admission to the hospital was mostly respiratory infections (37.3%), followed by infective endocarditis (16.9%), and central nervous system infections (13.6%). None of the patients had candidemia at admission. Among patients initially hospitalized for bacterial infections, candidemia developed after a median of 18 days (IQR: 12–34.5) from admission. *Candidozyma auris*-positive blood cultures were retrieved after a significantly longer period (*p* = 0.001), with a median of 30 days (IQR: 16.5–79.75, 95% C.I. = 29.83–59.25 days), while *Candida* spp.-positive cultures occurred after a median period of 15 days (IQR: 8–27.5, 95% C.I. = 13.22–23.00 days).

Regarding *Candida* species distribution, *Candidozyma auris* accounted for 24 patients (39.3%), while the remaining episodes were caused by *Candida albicans* (11 patients, 18%), *Candida glabrata* (now classified as *Nakaseomyces glabratus* [14], 16.4%), *Candida parapsilosis* (8 patients, 13.3%), *Candida krusei* (now classified as *Pichia kudriavzevii* [15], 8.2%), *Candida* tropicalis (4 patients, 6.6%) and *Candida kefyr* (1 case, 1.6%).

**Figure 1:**
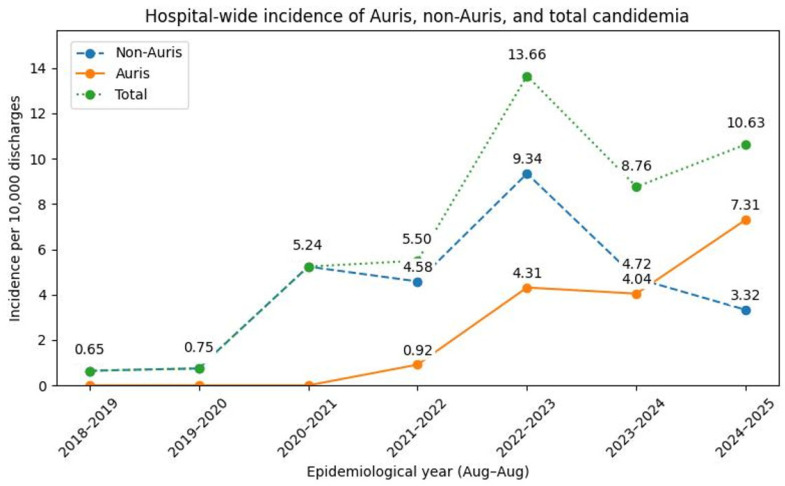
Hospital-wide incidence reported as10,000 discharges per epidemiological study year. Hospital-wide incidence of *Candidozyma auris*, *Candida* spp., and total candidemia cases per 10,000 hospital discharges across epidemiological study years (1 August–31 July).

Throughout the study period, the distribution of positive *Candida* blood cultures by epidemiological year (1 August to 31 July) and the annual proportion of *Candidozyma auris* versus *Candida* spp. species are summarized in Table 1. The absolute number of *Candidozyma auris* cases showed a marked increase over the epidemiological years, rising from 0 cases (2018–2021) to 11 cases in the final year of the study. This upward trend was concordant with the hospital-wide incidence per 10,000 discharges, as illustrated in Figure 1.

Risk factors for invasive candidiasis are presented in Table 2. Almost all patients had antibiotic therapy before candidemia, with no pattern observed between the antibiotics used and the type of *Candida* that was isolated.

Healthcare-associated exposure was highly common. Half of the patients had a history of prior hospitalization (50.8%). All patients had at least one invasive device in place at the time of the first positive *Candida* blood culture, with central venous catheters present in 73.8% of cases.

The distribution of *Candidozyma auris* did not significantly differ between ICU and non-ICU wards [9/22 (40.9) vs. 15/39 (38.5%); Fisher’s exact test, *p* = 1.00; OR 1.11, 95% C.I. 0.38–3.22].

A detailed description of demographic features, comorbidities, and primary infection site, along with risk factors, is provided in Table 2.

Antifungal susceptibility testing was available for 56 of 61 isolates (91.8%), including all 24 *Candidozyma auris* cases (100%) and 32 of 37 *Candida* spp. infections (86.5%). Resistance patterns differed markedly between *Candidozyma auris* and *Candida* spp. across all major antifungal drug classes (Table 3).

Azole susceptibility demonstrated striking inter-species differences. Among *Candidozyma auris* isolates, 23 of 24 (95.8%) were resistant to azoles, with only 1 isolate (4.2%) remaining susceptible. No intermediate susceptibility was documented for *Candidozyma auris*. In contrast, *Candida* spp. species showed considerably lower resistance rates: 8 of 32 isolates (25.0%) were resistant, 20 (62.5%) were susceptible, and 4 (12.5%) exhibited intermediate susceptibility (*p* < 0.001 for resistance comparison between groups).

Polyene susceptibility patterns revealed a concerning profile for *Candidozyma auris*. All 24 *Candidozyma auris* isolates (100%) demonstrated reduced susceptibility to amphotericin B according to EUCAST breakpoints: 18 isolates (75.0%) exhibited intermediate susceptibility and 6 (25.0%) demonstrated resistance, with no fully susceptible isolates identified. This contrasted sharply with *Candida* spp. species, where 30 of 32 isolates (93.8%) were fully susceptible, 2 (6.3%) showed intermediate susceptibility, and none were resistant (*p* < 0.001 for resistance comparison).

Echinocandin susceptibility was overall more favorable. Among isolates tested for echinocandin susceptibility (53 total), resistance rates were moderate: 5 of 29 *Candida* spp. isolates (17.2%) and 2 of 24 *Candidozyma auris* isolates (8.3%) were resistant, with no significant difference between groups (*p* = 0.448). The majority of isolates remained susceptible: 24 of 29 *Candida* spp. (82.8%) and 22 of 24 *Candidozyma auris* (91.7%). No intermediate susceptibility results were documented for echinocandins in either group.

Multi-class resistance patterns were observed exclusively in *Candidozyma auris* infections. Azole–polyene co-resistance was documented in five *Candidozyma auris* isolates (20.8%) compared with none in the *Candida* spp. group (*p* = 0.011). Azole–echinocandin co-resistance occurred in two *Candidozyma auris* cases (8.3%) versus none in *Candida* spp. isolates (*p* = 0.181). Polyene–echinocandin co-resistance was identified in one *Candidozyma auris* isolate (4.2%) and none in the *Candida* spp. group (*p* = 0.429). Resistance to all three antifungal classes was identified in one *Candidozyma auris* patient (4.2%). Detailed susceptibility distributions, including susceptible, intermediate, and resistant categories for each drug class and co-resistance patterns, are presented in Table 3.

### 3.2. Laboratory Parameters at Diagnosis and at Day 7

At the moment of candidemia, the inflammatory profile was largely comparable between the two groups, with no statistically significant differences observed in terms of C-reactive protein (CRP) (*p* = 0.36), and procalcitonin (PCT) (*p* = 0.83). Similarly, other parameters (hemoglobin, platelets, creatinine) exhibited comparable distribution across the two groups (*p* > 0.05).

However, some outstanding differences were identified upon a closer evaluation of the hematological profile. However, within the normal range, White Blood Cells (WBCs) and neutrophil counts were lower in the Auris group compared to the *Candida* spp. group, resulting in a significantly lower neutrophil-to-lymphocyte ratio (NLR) in patients infected with *Candidozyma auris*.

Detailed results regarding hematological and biochemical markers at the moment of diagnosis are displayed in Table 4.

Out of the 61 patients included in the study, 16 died and 5 were transferred to other medical units within the first 7 days following their candidemia diagnosis. Consequently, paired laboratory data at diagnosis and day 7 were available for 40 patients, who were included in the analysis of laboratory parameter dynamics. Regarding the dynamics of laboratory parameters, no significant changes were observed from diagnosis to day 7 in either *Candidozyma auris* or *Candida* spp. group (*p* > 0.05 for all comparisons). This analysis was restricted to patients with available paired laboratory measurements at both time points.

Fungal–bacterial bloodstream infection was documented in 24 patients during the course of candidemia. Among these cases, 10 involved Gram-negative bacteria and 14 involved Gram-positive bacteria, with Gram-positive co-infections occurring predominantly in the *Candida* spp. group (12/14). In 15 of the 24 patients (62.5%), bacterial growth was detected in the same blood culture bottle as the *Candida* isolate.

Persistent positive blood cultures at day 7 were documented in 8 of 28 patients with available repeat blood cultures. Persistent candidemia was more frequent in the *Candidozyma auris* group compared with the *Candida* spp. group (6 vs. 2 patients, *p* = 0.05). Notably, all patients with persistent candidemia died during hospitalization.

### 3.3. Treatment and Clinical Course

Regarding treatment, anidulafungin was the first-choice antifungal agent (70.5% overall) and was used significantly more often in *Candidozyma auris* infections compared with *Candida* spp. patients (91.7% vs. 56.8%, *p* = 0.004). Treatment duration was also longer among *Candidozyma auris* patients (median 14 days, IQR = 5–25.5, 95% C.I. = 10.81–22.05 vs. 6 days, IQR = 3–14, 95% C.I. = 6–14, *p* = 0.018). Antifungal treatment was guided by susceptibility testing whenever available; in the absence of susceptibility results, therapy was adjusted empirically according to the clinical context and local practice. Detailed treatment characteristics patterns are provided in Table 5.

In addition, individuals with *Candidozyma auris* candidemia had a substantially prolonged pre-infection hospitalization (median 30 days, IQR = 16.5–79.75, 95% C.I. = 29.83–59.25, vs. median 15 days, IQR = 8–27.5, 95% C.I. = 13.22–23.00, *p* = 0.001) and a markedly longer total length of hospitalization (median 52.5 days, IQR = 29.5–74.25, 95% C.I. = 44.14–79.69 vs. median 20 days, IQR = 11–39, 95% C.I. = 20.14–33.65, *p* < 0.001).

### 3.4. Mortality and Survival Analysis

**Figure 2:**
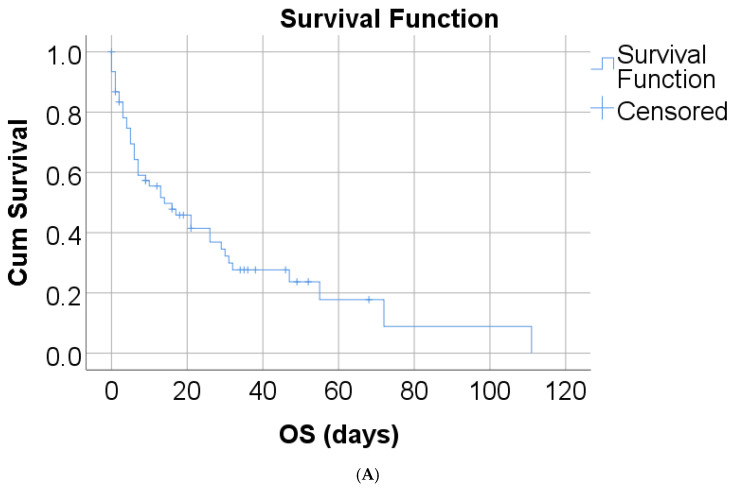

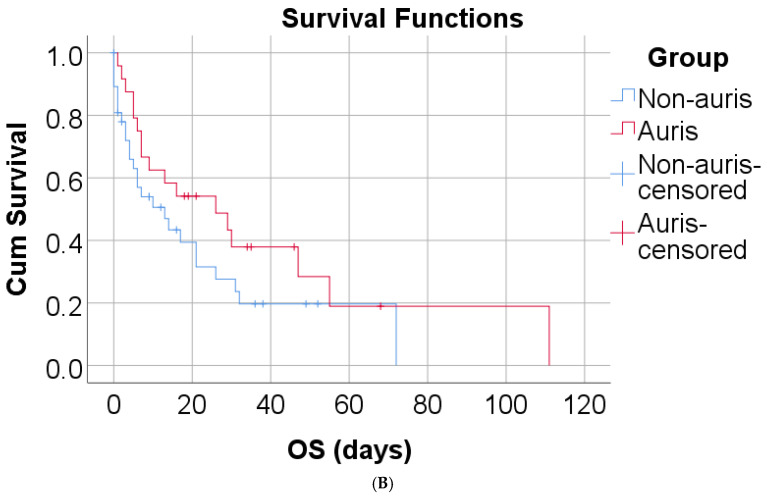
Kaplan–Meier survival analysis of patients with candidemia. + Censored patients are marked with crosses on the survival curve, representing individuals who did not reach the analyzed endpoint (all-cause in-hospital mortality) during the study period. Data for these patients were censored at the time of their last clinical contact, including hospital discharge or transfer to other medical units. OS = overall survival (days) (defined as time from positive blood culture to discharge or death, and all-cause in-hospital mortality). Cum Survival = Cumulative Survival. Log-rank test—*p* = 0.150. Univariable Cox-proportional hazard regression model for predicting mortality using patient group (*Candida* spp. vs. *Candidozyma auris*)—HR = 0.638; 95% C.I. = 0.340–1.195; *p* = 0.160. (**A**) Overall survival of the entire study cohort; (**B**) overall survival stratified by *Candidozyma auris* and *Candida* spp. candidemia.

Overall mortality was high, at 70.5% (43/61), with no significant difference between *Candidozyma auris* and *Candida* spp. groups (70.8% vs. 70.3%, *p* = 1.000). Median overall survival was 14 days (95% CI: 3.77–24.22), without a significant difference between groups (median OS = 26, 95% C.I. = 4.053–47.947 vs. median OS = 13, 95% C.I. = 2.926–23.074 days, *p* = 0.150), as illustrated in Figure 2A,B.

Factors significantly associated with mortality included mechanical ventilation (46.5% vs. 11.1%, *p* = 0.003) vasopressor requirements (55.8% vs. 11.8%, *p* = 0.003), and persistent candidemia (42.1% vs. 0%, *p* = 0.029). Among the laboratory parameters associated with mortality were lymphopenia < 500 cells/μL (39.5% vs. 5.6%, *p* = 0.021) and thrombocytopenia < 100,000/μL at diagnosis (44.2% vs. 5.6%, *p* < 0.001) and day 7 (55.6% vs. 15.4%, *p* = 0.025).

Mortality was substantially higher among patients managed in the ICU department, at 90.9%, compared with 59% for those treated in medical wards (*p* = 0.009). Overall survival was markedly reduced in the ICU group, with a median of 5 days (95% CI: 1.55–8.44), in contrast to 26 days (95% CI: 12.39–39.6) among non-ICU patients (*p* = 0.011).

### 3.5. Multivariate Analysis

Cox-proportional hazard regression demonstrated that thrombocytopenia was the only variable that remained independently associated with mortality (Table 6). In the multivariate model, only platelet counts below 100,000 cells/μL were associated with a more than two-fold increase in the risk of death (HR 2.34, 95% CI: 1.20–4.56; *p* = 0.012), underscoring the prognostic relevance of hematological dysfunction in this population.

## 4. Discussion

This single-center retrospective analysis provides a comprehensive, clinically grounded comparison between *Candidozyma auris* and *Candida* spp. bloodstream infection in a tertiary infectious diseases hospital. The single-center design also confers practical value: it ensured uniform case definitions, microbiological methods, and clinical management across the entire seven-year cohort, and it captured the local emergence of *Candidozyma auris* in real time. As one of the first head-to-head comparisons of *C. auris* and *Candida* spp. candidemia from this region, it provides other centers with a concrete reference—the incidence trajectory, the resistance profile, the predominance of non-ICU (ward) onset, and the prognostic value of thrombocytopenia—that can help calibrate local screening, infection-control, and empirical antifungal strategies in settings facing similar emergence of the pathogen. Over the seven-year study period, we observed a progressive increase in candidemia cases, noticeable by the emergence and subsequent rise in *Candidozyma auris.* Although *Candidozyma auris* candidemia developed after a significantly longer hospitalization period, required extended antifungal treatment and prolonged the total hospitalization period, risk factor profiles and mortality rates were comparable between the two groups.

This aspect reflects the ongoing shift among *Candida* species that causes bloodstream infections towards *Candidozyma auris*.

An increasing number of *Candida* isolates was observed in our cohort after 2020, coinciding with the COVID-19/post-COVID-19 pandemic, a pattern consistent with reports describing healthcare pressure during and after COVID-19 [16]. However, this trend is unlikely to be explained solely by the COVID-19 context and may also reflect a broader shift toward increased patient complexity, prolonged hospitalization, and sustained exposure to healthcare environments. This reflects the ongoing shift in bloodstream infection epidemiology toward *Candidozyma auris*. Our findings are consistent with reports from Romania, where *Candidozyma auris* has been recognized as an emerging hospital-associated pathogen and local outbreaks have been increasingly reported [17]. In Romania, the first cases of C auris were reported, similarly to our findings in 2022. The whole-genome sequencing revealed that all isolates belonged to the South Asian clade (clade I), suggesting clonal dissemination within and between healthcare facilities [18]. A subsequent analysis of 102 patients from 2022 to 2023 confirmed the ongoing spread of clade I strains with multiple resistance markers and reported a crude mortality rate of 68.18% among infected patients [18]. Our study contributes to this emerging body of evidence by providing a comparative analysis between *C. auris* and *Candida* spp. candidemia within a single infectious diseases hospital over seven years. Notably, we demonstrate that despite the extensive fluconazole resistance and prolonged hospitalization associated with *C. auris*, mortality rates were comparable to *Candida* spp. candidemia (70.8 vs. 70.3%), suggesting that patient-related factors and disease severity—rather than species-specific virulence—are the primary determinants of outcome.

The near-universal azole resistance observed in our *Candidozyma auris* cohort (95.8%) aligns with global surveillance data demonstrating fluconazole resistance rates exceeding 90% [19,20]. This intrinsic resistance is primarily mediated by mutations in the ERG11 gene encoding lanosterol 14-α-demethylase and overexpression of efflux pump genes CDR1 and MDR1 [21]. In contrast, the 25% azole resistance rate in *Candida* spp. likely reflects intrinsically resistant species such as *C. krusei* and acquired resistance in *C. glabrata* [22]. These findings support the exclusion of azoles as empirical therapy when *Candidozyma auris* is suspected or confirmed.

A particularly concerning finding was that all 24 *Candidozyma auris* isolates (100%) demonstrated reduced amphotericin B susceptibility: 75% intermediate and 25% resistant. In contrast, 93.8% of *Candida* spp. remained fully susceptible (*p* < 0.001). The intermediate category indicates potential efficacy with increased drug exposure to liposomal amphotericin B [9]. The 25% amphotericin B resistance in our *Candidozyma auris* cohort aligns with emerging reports from healthcare settings with sustained transmission [23,24]. This resistance is mediated by mutations in ergosterol biosynthesis genes (ERG3, ERG2, ERG6) [25]. The absence of fully susceptible *Candidozyma auris* isolates, combined with 95.8% azole resistance, positions echinocandins as the preferred first-line agents for *Candidozyma auris* candidemia [26].

Echinocandin resistance was numerically higher in *Candida* spp. species (17.2%) compared with *Candidozyma auris* (8.3%, *p* = 0.240). This likely reflects the species composition of the *Candida* spp. group, particularly *C. glabrata*, which demonstrates a well-documented propensity for developing echinocandin resistance through FKS1 and FKS2 mutations [27,28]. The overall echinocandin resistance rate of 13.2% exceeds that in many European surveillance reports and underscores the importance of routine susceptibility testing [29,30]. The preserved echinocandin susceptibility in *Candidozyma auris* (91.7%) supports current guideline recommendations positioning echinocandins as a first-line therapy for *Candidozyma auris* infections [9].

Multi-class resistance patterns were observed exclusively in *Candidozyma auris* infections, with no such patterns documented among *Candida* spp. species. Azole–polyene co-resistance affected 20.8% of *Candidozyma auris* isolates (5/24, *p* = 0.011), representing the predominant multi-class resistance pattern in our cohort. This combination severely restricts therapeutic options by simultaneously eliminating both azole and polyene classes, leaving echinocandins as the sole remaining first-line option. Azole–echinocandin co-resistance occurred in 8.3% of *Candidozyma auris* cases (2/24), while polyene–echinocandin co-resistance was documented in one isolate (4.2%).

Most critically, one patient in our cohort harbored an isolate resistant to all three major antifungal classes—azoles, polyenes, and echinocandins—representing an exceptionally challenging therapeutic scenario with severely limited standard treatment options. The management of such pan-resistant infections remains poorly defined, with case reports describing salvage approaches including combination therapy and investigational agents, though clinical outcome data are extremely limited [29].

The concentration of multi-class resistant isolates in our *Candidozyma auris* cohort (25% with resistance to ≥2 classes) is concerning and likely reflects several factors. *Candidozyma auris* demonstrates unique pathogenic features including enhanced biofilm formation and persistent adherence to healthcare surfaces, facilitating environmental contamination and patient colonization. These characteristics may contribute to selective pressure through repeated antifungal exposure in healthcare settings. Our findings underscore the critical importance of infection prevention and control measures, routine antifungal susceptibility testing, and antimicrobial stewardship to limit the emergence and spread of multi-resistant *Candidozyma auris* [31,32].

Both *Candidozyma auris* and *Candida* spp. candidemia developed after a considerable duration of hospitalization, underscoring their predominantly healthcare-associated nature. *Candida* spp. patients’ bloodstream infection in our cohort emerged after a median of 15 days from admission, whereas *Candidozyma auris* infections were diagnosed substantially later, with a median of 30 days. The delayed onset of *Candidozyma auris* bloodstream infection is consistent with prior colonization preceding invasive disease; however, clonal relatedness and the exact transmission pathway cannot be confirmed in the absence of molecular typing or environmental sampling. The process of colonization is facilitated by the organism’s high environmental persistence and ease of transmission within healthcare settings. These observations are consistent with the current infection-control policy recommendations in Romania, granted by the National Institute of Public Health, mirroring international policies that advise screening for colonization with *Candidozyma auris* in all patients with risk factors [1,4,33]. Documenting the day of diagnosis relative to admission for both groups further clarifies the temporal dynamics of acquisition and supports targeted interventions in high-risk units. Incidence was expressed per 10,000 hospital discharges because this denominator was consistently available across the full study period; however, patient-days, ICU-days, and device-utilization data were not available, which limits direct comparison with surveillance studies using exposure-adjusted denominator.

In our cohort, most candidemia episodes originated in medical wards, with 39 of 61 patients (63.9%), whereas only 22 patients (39.1%) were in intensive care units. The predominance of ward onset candidemia diverges from the traditional epidemiological pattern, where these infections were historically concentrated in critical care settings, and reflects a shift that is increasingly reported in new studies [1].

Several factors are likely contributors to this trend, including the growing complexity of non-ICU patients, prolonged hospitalization, broad-spectrum antibiotic use, and the extensive use of invasive devices in general wards. Device utilization was notably high across the cohort, with 73.8% of patients carrying a central venous catheter, 67.2% a urethro-vesical catheter, and 39.3% a nasogastric tube. This widespread exposure to invasive devices reflects the overall clinical complexity of the population, with critically ill patients being treated across all hospital units, not only in intensive care. Central venous catheters provide a direct portal for bloodstream invasion, while urinary and gastrointestinal instrumentation may facilitate mucosal disruption, promote local colonization and increase the risk of translocation [34,35,36]. These findings suggest that a strict catheter stewardship policy should be instated across both ICU and non-ICU patients.

The overall duration of in hospital stay was substantial in our cohort, with a median of 31 days, (IQR 16–54), exceeding the 22.2 days of hospitalization reported for invasive candidiasis in the United States of America [37]. Notably, hospital stay differed significantly between the two groups: *Candida* spp. candidemia occurred in patients hospitalized for a median of 20 days (IQR 11–39), whereas *Candidozyma auris* patients were characterized by a markedly prolonged length of stay, reaching a median of 52.5 days (IQR 29.5–74.25; *p* < 0.001). This pattern reinforces the broader epidemiological pattern observed throughout our analysis, in which *Candidozyma auris* affects individuals with prolonged healthcare exposure, reflecting its capacity for persistent colonization and its facile transmission within hospital settings. These findings have important infection prevention and control implications, as the emergence of *Candidozyma auris* in patients with prolonged hospitalization underscores the need for early screening, strict contact precautions, environmental decontamination, and enhanced diagnostic vigilance.

The inflammatory profile observed in our cohort revealed an overall low inflammatory systemic response with no significant differences between *Candidozyma auris* and *Candida* spp. candidemia in terms of WBC, CRP or PCT levels. This pattern aligns with the well-described characteristics of invasive candidiasis, where procalcitonin typically remains low or minimally elevated and C-reactive protein shows a moderate nonspecific rise [38,39]. The unexpectedly lower neutrophil counts and NLR observed in *Candidozyma auris* may reflect immune dysregulation and the frequent hematologic conditions found in this group (20.8%). In a study by İlker Ödemiş et al., an elevated NLR (median 9.2) was associated with increased mortality in candidemia; however, *Candidozyma auris* patients were not included in that analysis. In our cohort, the overall NLR was 7.48 (IQR 3.61–17.17), with *Candida* spp. patients exhibiting higher values-10.3% (IQR 4.3–23.4) compared with *Candidozyma auris* patients, who demonstrated significantly lower ratios at 4.7 (IQR 2.53–11.6, *p* = 0.020). These findings suggests that *Candidozyma auris* may present with a blunted inflammatory profile relative to other *Candida* species, warranting further evaluation of species-specific host responses [40,41]. Emerging evidence suggests that thrombocytes may play an active role in antifungal defense through interaction with *Candida* species, with experimental data suggesting that they can contribute, modestly, to antimicrobial activity [42,43]. In the neonatal population, thrombocytopenia is a well-described feature of invasive candidiasis and has even been incorporated as a supportive parameter in modified diagnostic scores [44]. In our analysis, thrombocytopenia emerged as the only independent predictor of mortality (HR 2.34, *p* = 0.012) even after adjustment for other clinical variables, while lymphopenia showed only an association in univariate analysis. These findings draw attention to the prognostic value of platelet counts in candidemia and suggest that close monitoring of thrombocyte levels may help identify patients at higher risk of poor outcomes, which could trigger early intensive therapeutic interventions.

The mortality rate in our cohort was remarkably high (70.5%), falling in the upper extreme of the ranges commonly reported in recently published articles [2,45]. When comparing the two groups there were no significant differences between *Candidozyma auris* and *Candida* spp. candidemia mortality: 70.8% vs. 70.3%, *p* =1.000. Median overall survival was 14 days (95% CI: 3.77–24.22), again with no meaningful difference between the two groups (26 vs. 13 days, *p* = 0.150). The substantially higher mortality observed among ICU patients (90.9% vs. 59% non-ICU) highlights the critically ill subgroups of patients in this department. A key observation emerging from our analysis is that, despite the recognized high transmissibility and environmental resilience of *C auris*, mortality did not differ from that observed in candidemia caused by other species. This suggests that once infection is established, patient outcomes are driven more by host factors and severity of illness rather than by species-specific virulence characteristics. After inclusion of *Candidozyma auris* versus *Candida* species type as a covariate in the multivariable model, thrombocytopenia remained the only independent predictor of mortality (HR 2.34, *p* = 0.012), while pathogen type was not significantly associated with outcome (HR 0.62, *p* = 0.145). Proportional hazards assumptions were confirmed for all variables retained in the final model. However, the model should be interpreted in the context of the retrospective data available, as validated severity scores, time to active antifungal therapy, and source-control measures were not consistently available.

The use of anidulafungin as the primary treatment in our cohort (70.5% overall and 91.7% among *Candidozyma auris* patients) is in line with the international guideline recommendations. This therapeutic approach corresponds to the 2016 IDSA guidelines, which endorsed echinocandins as first-line therapy for candidemia and were fully applicable to our study period, which started in 2018. Moreover, it remains consistent with the current ECMM/ISHAM recommendations, which continue to position echinocandins, especially anidulafungin, as the preferred initial agent for candidemia, particularly when suspecting *Candidozyma auris* infection, given its broad activity and favorable safety profile [9,26]. Notably, the extended treatment duration observed in *C. auris* infections (median 14 days) did not translate into a measurable survival benefit. This raises questions regarding the optimal therapeutic strategies, such as combination therapy. These findings emphasize the need for further research into tailored antifungal approaches and source control for this resilient pathogen.

Persistent candidemia represents a particularly severe clinical phenotype. In our cohort, all patients with persistent positive blood cultures at day 7 died during hospitalization, highlighting persistent bloodstream infection as a marker of extremely poor prognosis. Day-7 laboratory and microbiological assessments likely reflect early treatment response under predominantly empirical antifungal therapy rather than a fully targeted management, reinforcing the importance of early clearance assessment. Persistent candidemia was observed in a small subset of patients, but no definite source was identified in the retrospective analysis; therefore, a causal role with a specific focus or retained device cannot be established. All recommended measures for the prevention and control of healthcare-associated infections were applied in accordance with local and international guidelines; however, in these critically ill, intensive-care-dependent patients, the removal of indwelling central venous catheters was frequently not feasible, and the documented antifungal resistance may have further contributed to the persistence of bloodstream infection [9,46].

Several limitations merit consideration. The retrospective study design limits causal interference and may introduce selection bias. As a single-center retrospective analysis, our results should be interpreted with caution and may not be generalizable to institutions with different patient populations, referral patterns, or infection-control practices. In addition, the availability of laboratory parameters and follow-up blood cultures at day 7 was limited, reflecting real-world clinical practice. Given the retrospective nature of the study, during the earlier phases of the study period, repeated blood cultures and systematic early clearance assessments were not consistently implemented, partly due to evolving international guideline recommendations and the relatively low incidence of invasive candidiasis in routine clinical care. A further limitation of this study is the inherent heterogeneity of the *Candida* spp. comparator group, which encompasses multiple *Candida* species with distinct virulence profiles, antifungal resistance patterns, and associated mortality risks. This heterogeneity may have masked species-specific effects and should be considered when interpreting the comparisons between groups [2,46,47]. The study identifies several research priorities; prospective multicenter studies should validate thrombocytopenia as a prognostic biomarker and evaluate intervention thresholds. Randomized control trials comparing monotherapy versus combination therapy, particularly for *Candidozyma auris*, are needed. The development of rapid diagnostic tools capable of early species identification and resistance detection, particularly PCR-based methods for *C*. *auris* screening, may substantially improve clinical outcomes by enabling earlier targeted therapy [48].

## 5. Conclusions

This 7-year comparative analysis distinguishes *Candidozyma auris* as a unique, highly resistant healthcare-associated pathogen. Notably, mortality remained comparably high in both *Candidozyma auris* and *Candida* spp., with thrombocytopenia—not pathogen identity—emerging as the only independent predictor of mortality. Persistent infection led to poor outcomes, with a significant disease burden observed outside the ICU. These findings underscore a critical need for interventions: rigorous infection control, empiric echinocandin therapy where *C. auris* is prevalent, and early risk stratification for patients with long hospital stays and indwelling devices. Although limited to a single center, the consistent case definitions, uniform microbiological methods, and standardized management across seven years render these findings a practical reference for institutions managing the emergence of *C. auris*, ultimately guiding local screening, infection-control, and empirical antifungal protocols.

## Figures and Tables

**Table 1:** Frequency of *Candida* spp. isolates from blood cultures reported per epidemiological study year.

Epidemiological Year Period: 1 August to 31 July	2018–2019	2019–2020	2020–2021	2021–2022	2022–2023	2023–2024	2024–2025
Total hospital discharges (*n*)	15,472	13,337	9551	10,913	13,913	14,841	15,045
*Candidozyma auris* (*n*)	0	0	0	1	6	6	11
*Candida* spp. (*n*)	1	1	5	5	13	7	5
Total candidemia cases (*n*)	1	1	5	6	19	13	16

Annual numbers of candidemia cases are presented in relation to the total number of hospital discharges during each epidemiological year, (*n*) = number of cases.

**Table 2:** Baseline demographic and clinical characteristics of patients with candidemia, stratified by *Candidozyma auris* and *Candida* spp.

Parameter	Total	*Candida* spp.	*Candidozyma auris*	*p*
N, %	61 (100%)	37 (60.7%)	24 (39.3%)	-
Age (median (IQR)) (95% C.I.)	66 (54–73.5) 59.25–66.85	68 (55–73) 58.29–68.03	65.5 (46.25–74.75) 56.34–69.41	0.97 *
Gender (male) (Nr., %)	45 (73.8%)	29 (78.4%)	16 (66.7%)	0.38 **
*Primary infection Site (Nr., %)*				
Digestive	9 (16.3%)	6 (17.1%)	3 (12.5%)	0.49 **
Endocarditis	10 (16.9%)	5 (14.3%)	5 (20.8%)	
Urinary	2 (3.4%)	1 (2.9%)	1 (4.2%)	
Respiratory	22 (37.3%)	16 (45.7%)	6 (25%)	
Central nervous system (CNS)	8 (13.6%)	3 (8.6%)	5 (20.8%)	
Unknown	8 (13.6%)	4 (11.4%)	4 (16.7%)	
*Comorbidities (Nr., %)*				
Diabetes mellitus	15 (24.6%)	7 (18.9%)	8 (33.3%)	0.23 **
Hematologic conditions	7 (11.5%)	2 (5.4%)	5 (20.8%)	0.10 **
Neoplasia	4 (6.6%)	4 (10.8%)	0 (0%)	0.15 **
Transplant—h	3 (4.9%)	1 (2.7%)	2 (8.3%)	0.56 **
Other conditions	21 (34.4%)	15 (40.5%)	6 (25%)	0.27 **
*Other risk factors (Nr., %)*				
Corticotherapy	21 (34.4%)	13 (35.1%)	8 (33.3%)	1.00 **
Bedridden	23 (37.7%)	11 (29.7%)	12 (50%)	0.18 **
*Invasive medical devices*				
Central venous catheter	45 (73.8%)	25 (67.6%)	20 (83.3%)	0.24 **
Urethro-vesical catheter	41 (67.2%)	22 (59.5%)	19 (79.2%)	0.16 **
Nasogastric tube	24 (39.3%)	8 (21.6%)	16 (66.7%)	0.001 **
Mechanical ventilation	22 (36.1%)	12 (32.4%)	10 (41.7%)	0.59 **
*Healthcare exposure history (last 30 days)*				
Previous hospitalizations	31 (50.8%)	17 (45.9%)	14 (58.3%)	0.43 **
Surgery	5 (8.2%)	3 (8.1%)	2 (8.3%)	1.000 **
Antibiotics	57 (93.4%)	34 (91.9%)	23 (95.8%)	1.000 **

Demographic characteristics, comorbidities and initial clinical presentation in the overall cohort and stratified by *Candida* species (*Candidozyma auris* and *Candida* spp. isolates). Patients with other conditions (N = 21) are as follows: 11 patients (52.4%) with SARS-CoV-2 infection, 6 patients (28.6%) with HIV infection and 4 patients (19%) with tuberculous meningitis. Note: Continuous variables are presented as a median (IQR) and compared using * Mann–Whitney U Test; categorical variables are expressed as counts (%) and compared using ** Fisher’s Exact Test. N = number of cases; IQR = interquartile range; 95% C.I. = 95% confidence intervals for means.

**Table 3:** Antifungal susceptibility patterns stratified by *Candida* species.

Antifungal Class/Category	Total (*n* = 56)	*Candida* spp. (*n* = 32)	*Candodozyma auris* (*n* = 24)	*p*-Value *
Azoles				
Susceptible (S)	21 (37.5%)	20 (62.5%)	1 (4.2%)	<0.001
Intermediate (I)	4 (7.1%)	4 (12.5%)	0 (0%)	0.129
Resistant (R)	31 (55.4%)	8 (25.0%)	23 (95.8%)	<0.001
Polyenes				
Susceptible (S)	30 (53.6%)	30 (93.8%)	0 (0%)	<0.001
Intermediate (I)	20 (35.7%)	2 (6.3%)	18 (75.0%)	<0.001
Resistant (R)	6 (10.7%)	0 (0%)	6 (25.0%)	0.004
Echinocandins ^†^				
Susceptible (S)	46 (86.8%)	24 (82.8%)	22 (91.7%)	0.448
Intermediate (I)	0 (0%)	0 (0%)	0 (0%)	-
Resistant (R)	7 (13.2%)	5 (17.2%)	2 (8.3%)	0.240
Co-resistance patterns ^‡^				
Azole + Polyene	5 (8.9%)	0 (0%)	5 (20.8%)	0.011
Azole + Echinocandin	2 (3.6%)	0 (0%)	2 (8.3%)	0.181
Polyene + Echinocandin	1 (1.8%)	0 (0%)	1 (4.2%)	0.429
All three classes	1 (1.8%)	0 (0%)	1 (4.2%)	0.429

Note: Susceptibility testing was available for 56/61 isolates (91.8%): 32/37 (86.5%) *Candida* spp. and 24/24 (100%) *Candidozyma auris*. Intermediate (I) susceptibility was not classified as resistance. * Fisher’s exact test. ^†^ Echinocandin susceptibility testing available for 53 isolates (29 *Candida* spp., 24 auris). ^‡^ Co-resistance defined as resistance (R) to the specified antifungal drug classes. N = number of cases.

**Table 4:** Comparative analysis of laboratory parameters between *Candida* spp. and *Candidozyma auris* groups at the moment of diagnosis.

Laboratory Parameters—(Nr., %/Median (IQR)) (95% C.I.)				
Parameter	Total	*Candida* spp.	*Candidozyma auris*	*p*
WBC	9200 (5000–13,350) 8643.71–13,099.56	11100 (5000–17,285) 9298.65–16,002.43	7950 (4450–10,000) 5964.5–10,293.84	0.05 *
Neutrophils	7700 (3550–12,650) 6993.09–11,246.26	8900 (3700–15,050) 7833.95–14,193.08	5250 (3525–8150) 4181.49–8218.51	0.03 *
Lymphocytes	800 (500–1300) 812.17–1200.19	700 (450–1100) 713.63–1257.67	1000 (500–1575) 753.49–1322.18	0.44 *
Hemoglobin	9.1 (8.2–10.95) 9.106–10.192	9.4 (8.2–11.1) 9.157–10.703	8.6 (8.2–9.65) 8.488–9.945	0.20 *
Platelets (PLT) (* 1000)	120 (54–226.5) 122.419–190.859	118 (63.5–193) 107.555–186.553	129 (27.25–253.5) 105.689–237.144	0.84 *
Creatinine	1.13 (0.7–1.75) 1.202–1.879	1.1 (0.71–1.65) 1.054–1.808	1.2 (0.7–2.55) 1.041–2.379	0.74 *
CRP	6.29 (3.65–11) 6.684–10.814	7.4 (2.6–15.95) 6.891–13.182	4.69 (3.87–9.1) 4.717–8.812	0.36 *
Procalcitonin	0.68 (0.27–2.82) 1.426–6.164	0.7 (0.35–2.75) 0.900–5.180	0.66 (0.11–6.4) 0–13.128	0.83 *
NLR	7.48 (3.61–17.75) 9.194–16.289	10.3 (4.3–23.4) 10.781–21.471	4.7 (2.53–11.6) 4.735–9.858	0.02 *

Note: Continuous variables are presented as median (IQR) and compared using * Mann–Whitney U Test; IQR = interquartile range, 95% C.I. = 95% confidence intervals for means. WBC = white blood cells, CRP = C-reactive protein, NLR = neutrophils-to-lymphocytes ratio.

**Table 5:** Treatment characteristic in the overall cohort and stratified by *Candidozyma auris* and *Candida* spp. isolates.

Parameter	Total	*Candida* spp.	*Candidozyma auris*	*p*
Treatment (Nr., %)				
Anidulafungin	43 (70.5%)	21 (56.8%)	22 (91.7%)	0.004 **
Fluconazole	9 (14.8%)	8 (21.6%)	1 (4.2%)	0.076 **
Voriconazole	9 (14.8%)	7 (18.9%)	2 (8.3%)	0.462 **
Itraconazole	1 (1.6%)	1 (2.7%)	0 (0%)	1.000 **
Posaconazole	1 (1.6%)	1 (2.7%)	0 (0%)	1.000 **
Caspofungin	4 (6.6%)	2 (5.4%)	2 (8.3%)	0.643 **
Amphotericin B	1 (1.6%)	1 (2.7%)	0 (0%)	1.000 **

Categorical variables were compared using the ** Fisher’s exact test; N = number of cases.

**Table 6:** Univariable and multivariable Cox-proportional hazard regression models for predicting mortality.

Parameter	Univariable		Multivariable	
	HR (95% C.I.)	*p*	HR (95% C.I.)	*p*
*Candidozyma auris* (Ref. = *Candida* spp. group)	0.63 (0.34–1.19)	0.160	0.62 (0.33–1.17)	0.145
ICU department	2.15 (1.16–3.96)	0.014	1.83 (0.96–3.49)	0.066
Mechanical ventilation	1.83 (0.99–3.38)	0.051	-	-
Vasopressor support	2.25 (1.22–4.16)	0.010	-	-
Lymphocytes < 500	2.56 (1.34–4.90)	0.004	-	-
Platelets < 100,000	2.83 (1.49–5.39)	0.001	2.34 (1.20–4.56)	0.012

The multivariable model excluded the following variables analyzed in the univariable models for the following reasons: mechanical ventilation—lack of significance in the univariable model; vasopressor support—multicollinearity issue with ICU admission; lymphocytes < 500—violation of proportional hazard assumption in the univariable model. The final multivariable model therefore retained three covariates: *Candidozyma auris* versus *Candida* spp. (Group), ICU admission, and thrombocytopenia. With 43 deaths among 61 patients and three covariates in the final model, the events-per-variable ratio was approximately 14, exceeding the commonly recommended minimum of 10 events per variable and indicating a low risk of model overfitting. Proportional-hazards assumptions were formally tested and met for all retained covariates. HR, hazard ratio; CI, confidence interval; ICU, intensive care unit.

## Data Availability

Data are contained in this article; further inquiries can be directed to the corresponding authors.

## Abbreviations

The following abbreviations are used in this manuscript:

VBH	“Dr. Victor Babes” Clinical Hospital for Infectious and Tropical Diseases
CRP	C-reactive protein
PCT	Procalcitonin
NLR	Neutrophil-to-lymphocyte ratio
CLSI	Clinical and Laboratory Standards Institute
EUCAST	European Committee on Antimicrobial Susceptibility Testing
MIC	Minimum inhibitory concentrations
ICU	Intensive care unit
CNS	Central nervous system
S	Susceptible
I	Intermediate
R	Resistant
WBC	White Blood Cell
PLT	Platelets
